# The role of hypertension and diabetes mellitus on the etiology of middle cerebral artery disease

**DOI:** 10.1002/brb3.2521

**Published:** 2022-03-20

**Authors:** Changqing Zhang, Zixiao Li, Liping Liu, Yuehua Pu, Xinying Zou, Hongyi Yan, Yuesong Pan, Xingquan Zhao, Yilong Wang, Yongjun Wang

**Affiliations:** ^1^ Department of Neurology Beijing Tiantan Hospital Capital Medical University Beijing China; ^2^ China National Clinical Research Center for Neurological Diseases Beijing China

**Keywords:** ischemic stroke, middle cerebral artery disease, risk factors

## Abstract

**Background:**

Ischemic stroke (IS) caused by middle cerebral artery (MCA) disease is the most common type of IS caused by intracranial artery disease in the Chinese population. Hypertension and diabetes mellitus are the common risk factors of cerebral small vessel disease and large artery atherosclerosis (LAA). However, little is known about whether hypertension and diabetes mellitus had different correlations with the small artery occlusion (SAO) and LAA etiology of MCA disease. Therefore, our aim was to identify the predictors of the etiology of MCA disease.

**Methods:**

We consecutively enrolled 967 patients with noncardiogenic IS in unilateral MCA territory. Vascular risk factors and the clinical–radiologic features of IS were analyzed. The etiology of IS were classified as SAO or LAA according to the Stop Stroke Study Trial of Org 10172 in Acute Stroke Treatment classification criteria. Multivariable logistic regression was used to identify the differences in the predictors between SAO and LAA etiology of MCA disease.

**Results:**

Multivariable logistic regression identified male and hypertension as the predictors of the SAO etiology of MCA disease, however diabetes mellitus, repeated transient ischemic attack before the stroke, gaze palsy, aphasia, headache at admission, and disability at discharge as the predictors of the LAA etiology of MCA disease.

**Conclusion:**

Hypertension and diabetes mellitus are related with the different etiology of MCA disease.

## Graphical abstract

Male and hypertension were the predictors of the small artery occlusion etiology of middle cerebral artery disease; however, diabetes mellitus, repeated transient ischemic attack before the stroke, gaze palsy, aphasia, headache at admission, and disability at discharge were the predictors of the large artery atherosclerosis etiology of middle cerebral artery disease. Therefore, hypertension and diabetes mellitus are related with the different etiology of middle cerebral artery disease.

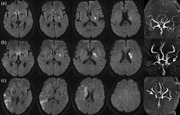

## 1 INTRODUCTION

Middle cerebral artery (MCA) is the most common location of intracranial artery disease in the Chinese population (Pu et al., 2013), so ischemic stroke (IS) caused by MCA disease is also the most common type of IS caused by intracranial artery disease. At present, little is known about the differences in the predictors between small artery occlusion (SAO) and large artery atherosclerosis (LAA) subtype of IS caused by MCA disease. Hypertension and diabetes mellitus are the common risk factors of cerebral small vessel disease and LAA. However, little is known about whether hypertension and diabetes mellitus had different correlation with the SAO and LAA etiological subtype of MCA disease. Therefore, the purpose of this study was to identify whether hypertension and diabetes mellitus had different correlation with the SAO and LAA etiological subtype of MCA disease.

## 2 SUBJECTS AND METHODS

### 2.1 Subjects

Chinese IntraCranial AtheroSclerosis Study (CICAS) is a prospective, multicenter, hospital‐based study. From October 2007 to June 2009, 2864 patients with noncardioembolic IS or transient ischemic attack (TIA) in 22 Chinese general hospitals were enrolled.

**FIGURE 1:**
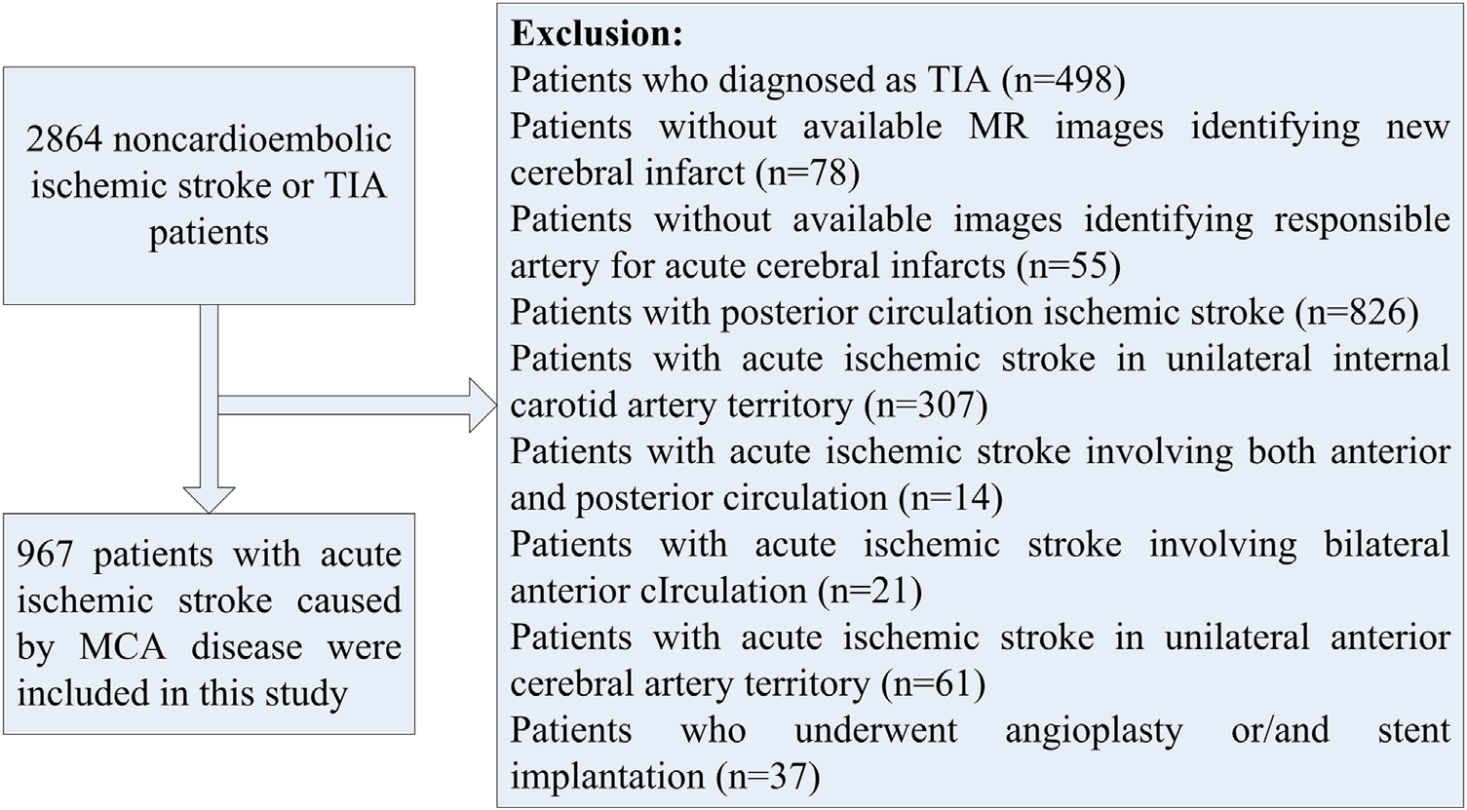
Flow chart of patient enrollment

Patients enrolled had the onset of symptoms within 7 days and were between 18 and 80 years old. Patients were excluded if they were clinically unstable, required close monitoring, unable to comply with magnetic resonance imaging (MRI). We excluded patients with cardioembolic risk factors (atrial fibrillation, atrial flutter, valvular heart disease, bioprosthetic or mechanical heart valve replacement, myocardial infarct within the past month, sick sinus syndrome, dilated cardiomyopathy, endocarditis, etc.) or other causes of IS as well as undetermined causes. Patients who were diagnosed as TIA, patients without available MR images identifying new cerebral infarct or responsible artery of acute infarcts, patients with IS involving posterior circulation or unilateral internal carotid artery territory or unilateral anterior cerebral artery territory or bilateral anterior circulation, patients with IS involving both anterior and posterior circulation, and patients who underwent angioplasty or stent implantation of intracranial or extracranial artery were also excluded. Finally, 967 patients with noncardiogenic IS in unilateral MCA territory were enrolled (Figure 1). The study protocol was approved by the ethics committee of Beijing Tiantan Hospital, and all participants or their legal proxies signed written informed consent for involvement of the research.

Clinical information including hypertension, diabetes mellitus, hyperlipidemia, history of coronary heart disease (CHD), smoking history, and heavy drinking history were defined according to the methods in previous paper (Pu et al., 2013). Primary symptoms and signs at admission, National Institutes of Health stroke scale score at admission and discharge, modified Rankin Scale (mRS) at discharge were also recorded. Disability was defined as mRS ≥2.

The data that support the findings of this study are available on request from the corresponding author. The data are not publicly available due to privacy or ethical restrictions.

### 2.2 MRI analysis

All patients underwent MRI including three‐dimensional time‐of‐flight MR angiography (MRA), axial T2‐weighted, T1‐weighted imaging, fluid‐attenuated inversion recovery sequences, and diffusion weighted imaging (DWI).

MCA was confirmed as the responsible artery when acute infarcts located in unilateral MCA territory, or acute borderzone infarcts were caused by ≥50% degree of stenosis or occlusion in the M1 segment of ipsilateral MCA, and there was no stenosis in the ipsilateral carotid artery. The degree of intracranial artery on MRA was calculated using the published method for the Warfarin–Aspirin Symptomatic Intracranial Disease Study (Samuels et al., 2000). The degree of extracranial artery stenosis was estimated by ultrasonography according to the published diagnostic criteria (Grant et al., 2003) or according to the North American Symptomatic Carotid Endarterectomy Trial criteria by contrast‐enhanced magnetic resonance angiography (CEMRA) (Fox, 1993).

**FIGURE 2:**
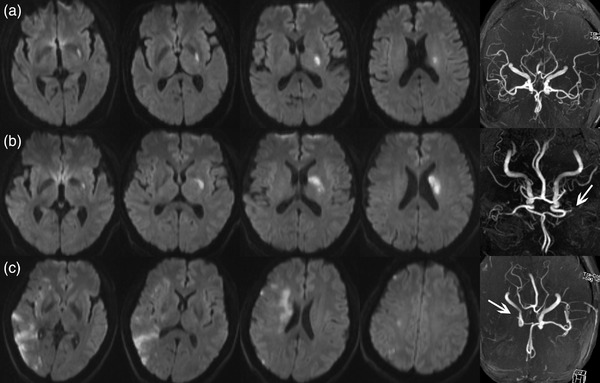
Topographical distribution of acute ischemic stroke caused by middle cerebral artery (MCA) disease. (a) Single perforating artery infarct in the left lenticulostriate artery territory, without the stenosis of left MCA. Small artery occlusion was the most probable etiology. (b) Single perforating artery infarct with occlusion of left MCA. Large artery atherosclerosis (LAA) and parent artery occluding penetrating artery were considered as the etiology and stroke mechanism, respectively. (c) Multiple acute infarcts (including internal borderzone infarcts, cortical borderzone infarcts, and small cortical infarct) in the right MCA territory, with the occlusion of right MCA. LAA and artery–artery embolism were considered as the etiology and stroke mechanism, respectively

The topographical distribution of acute infarcts (including single or multiple acute infarcts, borderzone infarcts, small cortical infarct, territorial infarct, and single perforating artery infarct) and etiological subtypes of IS were evaluated (Figure 2) (Zhang et al., 2019). Multiple acute cerebral infarcts were defined as ≥2 separate lesions that were hyperintense on DWI. Single perforating artery infarct was defined as a single infarct in the lenticulostriate artery territory. Borderzone infarcts were defined as the infarcts located at the junction of two (or three) artery territories with arterial collateral circulation. Borderzone infarcts were classified as internal borderzone infarcts and cortical borderzone infarcts. Internal borderzone infarcts were defined as rosary‐like pattern of infarcts arranged in a linear fashion parallel to the lateral ventricle and located in the centrum semiovale or corona radiata. Cortical borderzone infarcts are distinguished as anterior cortical borderzone infarcts and posterior cortical borderzone infarcts. Small cortical infarct was defined as the cortical infarct with a maximum diameter of <2 cm, excluding cortical borderzone infarct. Territorial infarct was defined as a large ischemic lesion with a maximum diameter of ≥2 cm involving the cerebral cortical and subcortical structure in one or more major cerebral artery territories. The etiological subtypes of IS were classified according to the Stop Stroke Study Trial of Org 10172 in Acute Stroke Treatment classification criteria. The stroke mechanism of LAA subtype of IS was determined as parent artery occluding penetrating artery if there was a single acute infarct located in the penetrating artery territory accompanied by any degree of stenosis in the parent artery; artery‐to‐artery embolism, when single or multiple small cortical infarcts, borderzone infarcts, or territory infarcts were caused by the stenosis of MCA; and multiple mechanisms, when two above mechanisms were present simultaneously (S. Gao et al., 2011).

Two radiologists blinded to the clinical details read all MRI scans. Consensus was reached by them if they had disagreement on interpretations.

### 2.3 Statistics

Mann–Whitney *U* test was used for comparison of continuous variables with non‐normal distribution, χ^2^ test was used for comparison of categorical variables. Multivariable logistic regression was used to identify the predictors of the SAO versus LAA etiology of MCA disease. All parameters that were significant on univariate analysis with *p* < .05 or likely to have pathophysiological influence were included in the multivariable regression analysis. All probability values were two‐tailed; *p* < .05 was considered statistically significant. All analyses were performed by using SAS Version 9.1 (SAS Institute, Cary, NC).

## 3 RESULTS

### 3.1 General patient characteristics

We analyzed 967 patients with noncardiogenic IS caused by MCA disease. A total of 405 patients (41.9%) and 562 patients (58.1%) were diagnosed as the SAO and LAA subtype of IS, respectively. As for vascular risk factors, 748 patients (77.4%) had hypertension and 307 (31.7%) had diabetes mellitus. Among 307 patients with diabetes mellitus, 268 patients had hypertension, while 39 patients did not (Table 1). Admission symptoms and signs, and imaging features of IS are presented in Table 2.

**TABLE 1:** Demographic features and vascular risk factors of 967 acute ischemic stroke caused by SAO or LAA etiology of middle cerebral artery disease

Variables	Total (*n* = 967)	SAO (*n* = 405)	LAA (*n* = 562)	*p*
**Demographics**				
Age, median (IQR), years	61 [52,71]	61 [52,71]	61 [52,71]	.935
Age ≥65 years	405 (41.9)	161 (39.8)	244 (43.4)	.255
Male	656 (67.8)	292 (72.1)	364 (64.8)	.016
**Vascular risk factors**				
Smoking	392 (40.5)	169 (41.7)	223 (39.7)	.522
Heavy drinking	50 (5.2)	24 (5.9)	26 (4.6)	.368
Hypertension	748 (77.4)	325 (80.2)	423 (75.3)	.068
Left SBP at admission (IQR), mmHg	150 [135,168]	150 [139,170]	146 [130,162]	.028
Right SBP at admission (IQR), mmHg	150 [135,169]	150 [135,170]	150 [132,161]	.023
Left DBP at admission (IQR), mmHg	90 [80,97]	90 [80,100]	86 [80,95]	.008
Right DBP at admission (IQR), mmHg	90 [80,98]	90 [80,100]	89 [80,95]	.056
Diabetes mellitus	307 (31.7)	112 (27.7)	195 (34.7)	.020
Diabetes mellitus without hypertension	39 (12.7)	13 (11.6)	26 (13.3)	.662
Hyperlipidemia	735 (76.0)	312 (77.0)	423 (75.3)	.525
Coronary heart disease	59 (6.1)	26 (6.4)	33 (5.9)	.725
History of ischemic stroke	222 (23.0)	79 (19.5)	143 (25.4)	.030

*Note*: Data are *n* (%) unless otherwise indicate.

DBP, diastolic blood pressure; IQR, interquartile range; LAA, large artery atherosclerosis; SAO, small‐artery occlusion; SBP, systolic blood pressure.

**TABLE 2:** Clinical and imaging features of 967 patients with acute ischemic stroke caused by middle cerebral artery disease

Variables	Total (*n* = 967)	SAO (*n* = 405)	LAA (*n* = 562)	*p*
**Admission symptoms and signs**				
Decreased alertness	44 (4.6)	5 (1.2)	39 (6.9)	<.0001
Gaze palsy	51 (5.3)	6 (1.5)	45 (8.0)	<.0001
Facial palsy	687 (71.0)	281 (69.4)	406 (72.2)	.333
Unilateral limb weakness	581 (60.1)	241 (59.5)	340 (60.5)	.756
Limb ataxia	76 (7.9)	40 (9.9)	36 (6.4)	.048
Sensory loss	303 (31.3)	123 (30.4)	180 (32.0)	.583
Aphasia	275 (28.4)	58 (14.3)	217 (38.6)	<.0001
Dysarthria	454 (46.9)	199 (49.1)	255 (45.4)	.247
Neglect	14 (1.4)	1 (.2)	13 (2.3)	.008
Dysphagia	64 (6.6)	24 (5.9)	40 (7.1)	.462
Headache	43 (4.4)	10 (2.5)	33 (5.9)	.011
Prestroke mRS, median (IQR)	0 [0,0]	0 [0,0]	0 [0,0]	.111
Admission NIHSS, median (IQR)	4 [2,8]	3 [2,6]	5 [2,9]	<.0001
Admission NIHSS ≤3	403 (41.7)	205 (50.6)	198 (35.2)	<.0001
Discharge NIHSS, median (IQR)	2 [1,5]	2 [1,3]	3 [1,6]	<.0001
Discharge mRS, median (IQR)	1 [1,3]	1 [1,2]	2 [1,3]	<.0001
Discharge mRS ≥2	469 (48.5)	154 (38.0)	315 (56.0)	<.0001
Repeated TIA before the stroke	52 (5.4)	18 (4.4)	34 (6.0)	.275
**Imaging features**				
Multiple acute infarcts	386 (39.9)	5 (1.2)	381 (67.8)	<.0001
Single perforating artery infarct	572 (59.2)	400 (98.8)	172 (30.6)	<.0001
Borderzone infarcts	358 (37.0)	0 (0)	358 (63.7)	<.0001
Internal borderzone infarcts	319 (33.0)	0 (0)	319 (56.8)	<.0001
Anterior cortical borderzone infarcts	188 (19.4)	0 (0)	188 (33.5)	<.0001
Posterior cortical borderzone infarcts	215 (22.2)	0 (0)	215 (38.3)	<.0001
Territorial infarcts	159 (16.4)	0 (0)	159 (28.3)	<.0001
Small cortical infarct	286 (29.6)	0 (0)	286 (50.9)	<.0001
Stenosis of MCA M1 segment ≥70%	470 (48.6)	0 (0)	470 (83.6)	<.0001

*Note*: Data are *n* (%) unless otherwise indicate.

IQR, interquartile range; LAA, large artery atherosclerosis; MCA, middle cerebral artery; mRS, the Modified Rankin Scale; NIHSS, National Institutes of Health stroke scale; SAO, small‐artery occlusion; TIA, transient ischemic attack.

### 3.2 Predictors of the SAO versus LAA etiology of MCA disease

Univariate analysis demonstrated that compared to the SAO group, the LAA group more often had diabetes mellitus and a history of IS. Regarding the clinical manifestations, the LAA group more commonly had decreased alertness, gaze palsy, aphasia, neglect, and headache, while less commonly had limb ataxia at admission. However, the SAO group more often were male and more frequently had a higher systolic blood pressure (SBP) at admission. There were no significant differences in the prevalence of hypertension between the SAO and the LAA group among these patients with diabetes mellitus. Multivariable logistic regression identified male (OR, 1.382; 95% CI, 1.027 to 1.858; *p* = .032) and hypertension (OR, 1.690; 95% CI, 1.207 to 2.368; *p* = .002) as the predictors of the SAO etiology of MCA disease, however, diabetes mellitus (OR, 0.697; 95% CI, 0.514 to 0.944; *p* = .020), repeated TIA before the stroke (OR, 0.464; 95% CI, 0.250 to 0.861; *p* = .015), gaze palsy (OR, 0.358; 95% CI, 0.140 to 0.915; *p* = .032), aphasia (OR, 0.331; 95% CI, 0.232 to 0.471; *p* < .0001), headache at admission (OR, 0.562; 95% CI, 0.338 to 0.934; *p* = .026), and disability at discharge (OR, 0.583; 95% CI, 0.422 to 0.806; *p* = .001) as the predictors of the LAA etiology of MCA disease (Table 3).

**TABLE 3:** Multivariable logistic regression for the predictors of the SAO versus LAA etiology of MCA disease

Variables	OR (95% CI)	*p*
Age ≥65 years	0.921 (0.686–1.236)	.584
Male	1.382 (1.027–1.858)	.032
Smoking	0.867 (0.622–1.208)	.399
Drinking	1.194 (0.637–2.237)	.580
Hypertension	1.690 (1.207––2.368)	.002
Diabetes mellitus	0.697 (0.514–0.944)	.020
Hyperlipidemia	1.198 (0.872–1.646)	.265
Coronary heart disease	1.093 (0.615–1.944)	.761
History of ischemic stroke	0.769 (.545–1.084)	.134
Repeated TIA before the stroke	0.464 (0.250–0.861)	.015
Admission NIHSS ≤3	1.072 (0.767–1.500)	.683
Discharge mRS ≥2	0.583 (0.422–0.806)	.001
Decreased alertness	0.516 (0.182–1.461)	.213
Gaze palsy	0.358 (0.140–0.915)	.032
Limb Ataxia	1.525 (0.922–2.521)	.100
Aphasia	0.331 (0.232–0.471)	<.0001
Neglect	0.178 (0.021–1.475)	.110
Headache	0.562 (0.338–0.934)	.026

CI, confidence interval; LAA, large artery atherosclerosis; MCA, middle cerebral artery; mRS, the Modified Rankin Scale; NIHSS, National Institutes of Health stroke scale; OR, odds ratio; SAO, small‐artery occlusion; TIA, transient ischemic attack.

## 4 DISCUSSION

The proportion of ischemic cerebrovascular disease caused by intracranial atherosclerosis in the Chinese population is much higher than in the Western White population (Pu et al., 2013; White et al., 2005). Our previous study also found IS in the MCA territory is far more common than IS in the internal carotid artery territory (Zhang et al., 2019).

MCA is the most common location of intracranial artery disease in the Chinese population (Pu et al., 2013); therefore, IS caused by MCA disease is also the most common type of IS caused by intracranial artery disease. In this study, we demonstrated the SAO subtype of MCA disease more frequently had a higher systolic blood pressure at admission, while the LAA subtype of MCA disease more frequently had diabetes mellitus. Hypertension was the predictor of the SAO etiology of MCA disease; however, diabetes mellitus was the predictor of the LAA etiology of MCA disease. These findings suggest that hypertension has a stronger correlation with SAO instead of LAA, while diabetes mellitus has a closer correlation with LAA instead of SAO. Therefore, hypertension and diabetes mellitus are associated with different etiology of MCA disease.

Previous studies found hypertension was the risk factor of both silent brain infarction (SBI) and symptomatic lacunar infarction (sLAC), while diabetes mellitus was only the risk factor of sLAC (Kim et al., 2011). We found the LAA group more frequently had serious clinical manifestations than the SAO group. Therefore, more sLAC patients might be the LAA subtype in the above study; however, more SBI patients might be the SAO subtype. The SAO subtype of IS belongs to the category of cerebral small vessel disease. Hypertension is the most important risk factor for cerebral small vessel disease (Filomena et al., 2015). But compared with hypertension, the correlation between diabetes mellitus and cerebral small vessel disease is not very definite (Vermeer et al., 2007). In our CICAS study, 33.8% (967/2864) IS were caused by MCA disease. Among them, the SAO group accounted for 41.9%, and 77.4% patients with MCA disease had hypertension. These percentages are all very high. Our study found the SAO group more frequently had a higher systolic blood pressure at admission than the LAA group. Therefore, we speculated the higher prevalence and lower control rate of hypertension may be responsible for the higher prevalence of MCA disease in the Chinese populations (Danaei et al., 2011; Wang et al., 2018; White et al., 2005), especially for the higher prevalence of the SAO subtype of MCA disease, in spite of the variations in genetic susceptibility may also be a possible cause (Saposnik et al., 2003; White et al., 2005). Therefore, improving the control rate of hypertension may reduce the incidence of MCA disease in the Chinese population.

The SAO and LAA subtype of MCA disease accounted for 41.9% and 58.1% in our study, respectively. Our study also found that single perforating artery infarct was the most common infarction pattern caused by MCA disease, and the most common etiology of single perforating artery infarct was SAO, followed by LAA. We found 98.8% (400/405) patients in the SAO group had single perforating artery infarct, and only five patients (1.2%) had multiple and simultaneous perforating artery infarcts in ipsilateral lenticulostriate artery territory. Previous researches reported that patients with acute simultaneous multiple lacunar infarcts (MLI) had a higher recurrence risk and a higher proportion of disability compared to acute single lacunar infarct (SLI). Hypertension was found to be more prevalent, and admission SBP and diastolic blood pressure were found to be higher in MLI patients (Ohara et al., 2005; Spolveri et al., 1998). Therefore, MLI may be a more severe entity of small artery disease compared to SLI (Lee et al., 2012). Fibrinoid necrosis or hyalinosis caused by hypertension was thought to be the underlying vasculopathy of MLI (Ohara et al., 2005). Simultaneous bilateral hypertensive putamen or thalamus hemorrhage was also reported, and simultaneous rupture of microaneurysms in the perforating arteries by the sharply increased blood pressure was believed to be the probable etiology of symmetric hemorrhages simultaneously (Kono & Terada, 2014). Arteriolosclerosis is strongly associated with hypertension, its main pathological features include fibrinoid necrosis, hyalinosis, microatheroma, and microaneurysms and can result in ischemic consequences (i.e., leukoaraiosis and lacunar lesions) and hemorrhagic lesions of the brain parenchyma (i.e., microbleeds and large hematoma in basal ganglia) (Pantoni, 2010). Since the sharp increase of blood pressure can lead to the simultaneous rupture of microaneurysms in bilateral lenticulostriate arteries and the secondary cerebral hemorrhage in bilateral basal ganglia, and then it can also lead to the simultaneous fibrinoid necrosis or hyalinosis of multiple lenticulostriate arteries and the secondary acute MLI. Therefore, hypertension was believed to be the main reason for multiple and simultaneous lenticulostriate artery infarcts in the SAO group of our study.

Diabetes mellitus was reported to be related to the severity and progression of white matter hyperintensities (Gouw et al., 2008; Lucatelli et al., 2016). However, many large‐scale observational studies did not find the significant correlation between diabetes mellitus and the incidence of SBI (Gouw et al., 2008; Vermeer et al., 2002). Therefore, whether diabetes mellitus was a risk factor of SBI or the SAO subtype of IS remains unclear (Vermeer et al., 2007). However, diabetes mellitus was an important risk factor of intracranial arteriosclerosis. Intracranial stenosis was approximately 3.13 times more frequently observed in the diabetic population than in the non‐diabetic population (Mendes et al., 1999). Compared to non‐diabetic patients with recent lacunar stroke, diabetic patients with recent lacunar stroke more frequently had intracranial stenosis ≥50% (Palacio et al., 2014). High‑resolution magnetic resonance imaging also demonstrated that patients with the SAO subtype of IS had a higher prevalence of hypertension compared with the patients with LAA subtype of IS (80% versus 29%; *p* < .001); however, patients with the LAA subtype of IS had a higher prevalence of diabetes mellitus compared with the patients with the SAO subtype of IS (40% versus 15%; *p* = .054) (T. Gao et al., 2014). In diabetic patients, insulin deficiency often causes glucose to convert into a large amount of fat, which results in hyperlipidemia and LAA. However, the main pathological features of SAO or arteriolosclerosis are fibrinoid necrosis and hyalinosis, which are most often caused by hypertension. Therefore, hypertension is the most widely accepted risk factor for SBI or SAO subtype of IS. Consequently, it is not hard to understand why diabetes mellitus has a closer correlation with LAA instead of SAO, while hypertension has a stronger correlation with SAO instead of LAA.

Our study had some limitations. First, this is a hospital‐based study, and patients who were clinically unstable, unable to comply with MRI were excluded, and these may possibly result in a selection bias. Second, high‐resolution MR was not performed; therefore, some patients in the SAO group may have a MCA plaque in the high‐resolution MR, although there are no abnormalities in the MRA at all. This is another limitation of our study.

## 5 CONCLUSIONS

Hypertension and diabetes mellitus are related with different etiology of middle cerebral artery disease.

## CONFLICT OF INTEREST

The authors declare that there is no conflict of interest that could be perceived as prejudicing the impartiality of the research reported.

### PEER REVIEW

The peer review history for this article is available at https://publons.com/publon/10.1002/brb3.2521

## Data Availability

The data that support the findings of this study are available on request from the corresponding author. The data are not publicly available due to privacy or ethical restrictions.

## References

[brb32521-bib-0001] Danaei, G. , Finucane, M. M. , Lin, J. K. , Singh, G. M. , Paciorek, C. J. , Cowan, M. J. , Farzadfar, F. , Stevens, G. A. , Lim, S. S. , Riley, L. M. , & Ezzati, M. (2011). National, regional, and global trends in systolic blood pressure since 1980: Systematic analysis of health examination surveys and epidemiological studies with 786 country‐years and 5.4 million participants. Lancet, 377, 568–577. 10.1016/S0140-6736(10)62036-3 21295844

[brb32521-bib-0002] Filomena, J. , Riba‐Llena, I. , Vinyoles, E. , Tovar, J. L. , Mundet, X. , Castañé, X. , Vilar, A. , López‐Rueda, A. , Jiménez‐Baladó, J. , Cartanyà, A. , Montaner, J. , & Delgado, P. (2015). Short‐term blood pressure variability relates to the presence of subclinical brain small vessel disease in primary hypertension. Hypertension, 66, 634–640; discussion 445. 10.1161/HYPERTENSIONAHA.115.05440 26101344

[brb32521-bib-0003] Fox, A. J. (1993). How to measure carotid stenosis. Radiology, 186, 316–318. 10.1148/radiology.186.2.8421726 8421726

[brb32521-bib-0004] Gao, S. , Wang, Y. J. , Xu, A. D. , Li, Y. S. , & Wang, D. Z. (2011). Chinese ischemic stroke subclassification. Frontiers in Neurology, 2, 6. 10.3389/fneur.2011.00006 21427797 PMC3052771

[brb32521-bib-0005] Gao, T. , Yu, W. , & Liu, C. (2014). Mechanisms of ischemic stroke in patients with intracranial atherosclerosis: A high‐resolution magnetic resonance imaging study. Experimental and Therapeutic Medicine, 7, 1415–1419. 10.3892/etm.2014.1600 24940449 PMC3991508

[brb32521-bib-0006] Gouw, A. A. , Van Der Flier, W. M. , Fazekas, F. , Van Straaten, E. C. W. , Pantoni, L. , Poggesi, A. , Inzitari, D. , Erkinjuntti, T. , Wahlund, L. O. , Waldemar, G. , Schmidt, R. , Scheltens, P. , & Barkhof, F. (2008). Progression of white matter hyperintensities and incidence of new lacunes over a 3‐year period: The leukoaraiosis and disability study. Stroke: A Journal of Cerebral Circulation, 39, 1414–1420. 10.1161/STROKEAHA.107.498535 18323505

[brb32521-bib-0007] Grant, E. G. , Benson, C. B. , Moneta, G. L. , Alexandrov, A. V. , Baker, J. D. , Bluth, E. I. , Carroll, B. A. , Eliasziw, M. , Gocke, J. , Hertzberg, B. S. , Katarick, S. , Needleman, L. , Pellerito, J. , Polak, J. F. , Rholl, K. S. , Wooster, D. L. , & Zierler, E. (2003). Carotid artery stenosis: Grayscale and Doppler ultrasound diagnosis—Society of Radiologists in ultrasound consensus conference. Ultrasound Quarterly, 19, 190–198. 10.1097/00013644-200312000-00005 14730262

[brb32521-bib-0008] Kim, M.‐H. , Moon, J.‐S. , Park, S.‐Y. , An, S.‐A. , Kim, O.‐J. , Kim, N.‐K. , & Oh, S.‐H. (2011). Different risk factor profiles between silent brain infarction and symptomatic lacunar infarction. European Neurology, 65, 250–256. 10.1159/000324335 21464570

[brb32521-bib-0009] Kono, K. , & Terada, T. (2014). Simultaneous bilateral hypertensive putaminal or thalamic hemorrhage: Case report and systematic review of the literature. Turkish Neurosurgery, 24, 434–437. 24848190 10.5137/1019-5149.JTN.8552-13.0

[brb32521-bib-0010] Lee, J. H. , Kim, Y. J. , Moon, Y. , Cho, H.‐J. , & Kim, H. Y. (2012). Acute simultaneous multiple lacunar infarcts: A severe disease entity in small artery disease. European Neurology, 67, 303–311. 10.1159/000336061 22517446

[brb32521-bib-0011] Lucatelli, P. , Montisci, R. , Sanfilippo, R. , Sacconi, B. , Suri, J. S. , Catalano, C. , & Saba, L. (2016). Is there an association between leukoaraiosis volume and diabetes? Journal of Neuroradiology, 43, 273–279. 10.1016/j.neurad.2015.11.003 26740385

[brb32521-bib-0012] Mendes, I. , Baptista, P. , Soares, F. , Oliveira, V. , & Ferro, J. M. (1999). Diabetes mellitus and intracranial stenosis. Reviews Neurology, 28, 1030–1033. 10390765

[brb32521-bib-0013] Ohara, T. , Yamamoto, Y. , Oiwa, K. , Hayashi, M. , & Nakagawa, M. (2005) Clinical classification for lacunar infarct. An investigation of 130 consecutive cases of lacunar infarctions. Rinsho Shinkeigaku = Clinical Neurology, 45, 6–12. 15714993

[brb32521-bib-0014] Palacio, S. , Mcclure, L. A. , Benavente, O. R. , Bazan, C. , Pergola, P. , & Hart, R. G. (2014). Lacunar strokes in patients with diabetes mellitus: Risk factors, infarct location, and prognosis: The secondary prevention of small subcortical strokes study. Stroke: A Journal of Cerebral Circulation, 45, 2689–2694. 10.1161/STROKEAHA.114.005018 PMC4146755 25034716

[brb32521-bib-0015] Pantoni, L. (2010). Cerebral small vessel disease: From pathogenesis and clinical characteristics to therapeutic challenges. Lancet Neurology, 9, 689–701. 10.1016/S1474-4422(10)70104-6 20610345

[brb32521-bib-0016] Pu, Y. , Liu, L. , Wang, Y. , Zou, X. , Pan, Y. , Soo, Y. , Leung, T. , Zhao, X. , Wong, K. S. , & Wang, Y. (2013). Geographic and sex difference in the distribution of intracranial atherosclerosis in china. Stroke: A Journal of Cerebral Circulation, 44, 2109–2114. 10.1161/STROKEAHA.113.001522 23760212

[brb32521-bib-0017] Samuels, O. B. , Joseph, G. J. , Lynn, M. J. , & Smith, H. A. , & Chimowitz, M. I. (2000). A standardized method for measuring intracranial arterial stenosis. AJNR. American Journal of Neuroradiology, 21, 643–646. 10782772 PMC7976653

[brb32521-bib-0018] Saposnik, G. , & Del Brutto, O. H.; Iberoamerican Society of Cerebrovascular D . (2003). Stroke in South America: A systematic review of incidence, prevalence, and stroke subtypes. Stroke: A Journal of Cerebral Circulation, 34, 2103–2107. 10.1161/01.STR.0000088063.74250.DB 12907823

[brb32521-bib-0019] Spolveri, S. , Baruffi, M. C. , Cappelletti, C. , Semerano, F. , Rossi, S. , Pracucci, G. , & Inzitari, D. (1998). Vascular risk factors linked to multiple lacunar infarcts. Cerebrovascular Diseases, 8, 152–157. 10.1159/000015841 9619698

[brb32521-bib-0020] Vermeer, S. E. , Koudstaal, P. J. , Oudkerk, M. , Hofman, A. , & Breteler, M. M. B. (2002). Prevalence and risk factors of silent brain infarcts in the population‐based Rotterdam Scan Study. Stroke: A Journal of Cerebral Circulation, 33, 21–25. 10.1161/hs0102.101629 11779883

[brb32521-bib-0021] Vermeer, S. E. , Longstreth, W. T. , & Koudstaal, P. J. (2007). Silent brain infarcts: A systematic review. Lancet Neurology, 6, 611–619. 10.1016/S1474-4422(07)70170-9 17582361

[brb32521-bib-0022] Wang, Z. , Chen, Z. , Zhang, L. , Wang, X. , Hao, G. , Zhang, Z. , Shao, L. , Tian, Y. , Dong, Y. , Zheng, C. , Wang, J. , Zhu, M. , Weintraub, W. S. , & Gao, R. (2018). Status of hypertension in China: Results from the China hypertension survey, 2012–2015. Circulation, 137, 2344–2356. 10.1161/CIRCULATIONAHA.117.032380 29449338

[brb32521-bib-0023] White, H. , Boden‐Albala, B. , Wang, C. , Elkind, M. S. V. , Rundek, T. , Wright, C. B. , & Sacco, R. L. (2005). Ischemic stroke subtype incidence among whites, blacks, and Hispanics: The Northern Manhattan Study. Circulation, 111, 1327–1331. 10.1161/01.CIR.0000157736.19739.D0 15769776

[brb32521-bib-0024] Zhang, C. , Wang, Y. , Zhao, X. , Liu, L. , Wang, C. , Li, Z. , Pu, Y. , Zou, X. , Pan, Y. , & Wang, Y. (2019). Clinical, imaging features and outcome in internal carotid artery versus middle cerebral artery disease. PLoS One, 14, e0225906 10.1371/journal.pone.0225906 31805111 PMC6894760

